# Exercise improves cardiac fibrosis by stimulating the release of endothelial progenitor cell-derived exosomes and upregulating miR-126 expression

**DOI:** 10.3389/fcvm.2024.1323329

**Published:** 2024-05-09

**Authors:** Genzhuo Fu, Zhao Wang, Siyuan Hu

**Affiliations:** ^1^ School of Medicine, Hunan University of Chinese Medicine, Changsha, China; ^2^ School of Traditional Chinese Medicine, Hunan University of Chinese Medicine, Changsha, China; ^3^ School of Sports and Arts, Hunan University of Chinese Medicine, Changsha, China

**Keywords:** exercise, exosomes, miR-126, cardiac fibrosis, TGF-β/Smad3

## Abstract

Cardiac fibrosis is an important pathological manifestation of various cardiac diseases such as hypertension, coronary heart disease, and cardiomyopathy, and it is also a key link in heart failure. Previous studies have confirmed that exercise can enhance cardiac function and improve cardiac fibrosis, but the molecular target is still unclear. In this review, we introduce the important role of miR-126 in cardiac protection, and find that it can regulate TGF-β/Smad3 signaling pathway, inhibit cardiac fibroblasts transdifferentiation, and reduce the production of collagen fibers. Recent studies have shown that exosomes secreted by cells can play a specific role through intercellular communication through the microRNAs carried by exosomes. Cardiac endothelial progenitor cell-derived exosomes (EPC-Exos) carry miR-126, and exercise training can not only enhance the release of exosomes, but also up-regulate the expression of miR-126. Therefore, through derivation and analysis, it is believed that exercise can inhibit TGF-β/Smad3 signaling pathway by up-regulating the expression of miR-126 in EPC-Exos, thereby weakening the transdifferentiation of cardiac fibroblasts into myofibroblasts. This review summarizes the specific pathways of exercise to improve cardiac fibrosis by regulating exosomes, which provides new ideas for exercise to promote cardiovascular health.

## 1 Introduction

According to the “Report on Cardiovascular Health and Diseases in China 2022”, cardiovascular disease is the leading cause of death among Chinese residents. Approximately 330 million individuals are afflicted with cardiovascular diseases, wherein hypertension, hyperlipidemia, and hyperglycemia emerge as primary etiological factors (1). In recent years, the incidence, hospitalization rate and mortality of cardiac fibrosis in diseases such as hypertension, coronary heart disease and cardiomyopathy have shown an upward trend, causing tremendous pain and economic burden to patients and their families. Therefore, analyzing the pathological mechanism of cardiac fibrosis and exploring safe and effective prevention and treatment strategies are important research topics in the field of cardiovascular medicine.

Research indicates that exercise can augment cardiac function and enhance the quality of life for patients with cardiovascular disease (1), however, the underlying mechanisms remain elusive. microRNAs have been implicated in crucial processes such as cardiomyocyte differentiation and transcription, playing a pivotal role in the regulation of cardiovascular disease (2, 3), notably, miR-126, miR-29, miR-24, and miR-21 exhibit close associations with cardiac fibrosis (4–7). Recent research indicates that physical exercise can enhance the release of exosomes and modulate the expression of miR-126 (8). We hypothesize that this phenomenon may be attributed to the potential of exercise in ameliorating cardiac fibrosis. This article systematically reviews the existing literature and summarizes the specific role of exosome-mediated microRNA-126 in the process of cardiac fibrosis. It also highlights the impact of exercise on the release of exosomes and the regulation of microRNA-126 expression. By synthesizing existing research findings, we hope to gain a deeper understanding of the specific molecular mechanisms underlying the improvement of cardiac fibrosis by exercise, thereby providing a more scientific theoretical basis and new ideas for the prevention and treatment of cardiovascular diseases.

## 2 Current status of treatment for cardiac fibrosis

Cardiac fibrosis denotes a pathological response characterized by persistent myocardial tissue damage, which triggers an excessive proliferation of fibroblasts, secretion of substantial amounts of collagen fibers, and subsequent excessive deposition (9). Furthermore, it can result in diminished cardiac compliance, necrosis of cardiomyocytes, and cardiac remodeling, ultimately leading to impaired myocardial contractility and cardiac dysfunction. These pathological changes serve as crucial links between hypertension, coronary heart disease, diabetes, and the development of heart failure (10–12). Cardiac fibroblasts represent the predominant population of mesenchymal cells within the cardiac tissue. Activation of profibrotic signaling pathways can induce their differentiation into myofibroblasts, leading to enhanced secretion of collagen fibers and subsequent scar formation. Notably, α-smooth muscle actin (α-SMA) serves as a reliable marker for discerning activated cardiac fibroblasts (13, 14). The studies suggest that TGF-β in the cardiac interstitium performs multiple functions, including the promotion of α-SMA transcription in fibroblasts through activation of Smad3 signaling (15). Additionally, it facilitates fibroblasts transdifferentiation and regulates the ratio of Col1 to Col3, thereby promoting the initiation and progression of cardiac fibrosis (16).

Cardiac fibrosis is regulated by a variety of factors, so there are different antifibrotic drugs for different regulatory factors in clinical practice. The overactivation of the renin-angiotensin-aldosterone system is the main mechanism leading to heart failure and cardiac fibrosis. Therefore, angiotensin converting enzyme inhibitors (ACEI), angiotensin receptor blockers (ARB), and aldosterone receptor antagonists have inhibitory effects on cardiac fibrosis. In addition, calcium ions promote fibroblasts proliferation, on this basis, calcium ion blockers are widely used in the treatment of fibrosis. Furthermore, pirfenidone is often used in clinical practice, and in addition to its antioxidant properties, it can also alleviate cardiac fibrosis by down-regulating TGF-β1 expression (17). Additionally, α1A-adrenergic receptor blockers have demonstrated the ability to attenuate the expression of TGF-β, p-Smad2, and p-Smad3 while effectively inhibiting excessive activation of cardiac fibroblasts (18). Furthermore, anti-inflammatory treatment has the potential to ameliorate cardiac fibrosis. For instance, colchicine exhibits anti-inflammatory properties while concurrently inhibiting fibrotic processes (19). The administration of Candesartan leads to a reduction in fibrosis through the downregulation of type III procollagen-N-peptide levels and inflammatory marker levels (20). In fact, some traditional Chinese medicines have also been shown to reduce the production of collagen fibers, such as “Shexiang Tongxin Drops” which can effectively inhibit the synthesis of α-SMA, Col1 and Col3 (21). However, most of the above methods involve exogenous administration of antagonists, anti-inflammatory drugs and other drugs to inhibit fibrosis, which leads to poor specificity, many adverse reactions, and unsatisfactory clinical effects. Therefore, it is imperative to clarify the pathological mechanism of cardiac fibrosis and explore direct targeted drugs to solve these challenges.

A number of therapeutic strategies targeting fibroblasts have emerged in recent studies. Such as cell reprogramming technology, it can directly induce fibroblasts into cardiac-like myocytes (iCLMs), which are expected to act as cardiomyocytes to directly enhance cardiac function (22). Besides, CAR-T immunotherapy has also been applied to the treatment of anti-fibrosis, using endogenous protein markers to directly target cardiac fibroblasts to optimize the therapeutic effect (23). Of course, cardiac repair through the activation of acute immune response by stem cells is also an important direction for the treatment of cardiac fibrosis and heart failure (24). In addition, recent studies have found that some drug results in the treatment of cardiovascular diseases have unexpected “off-target” effects on cardiac fibrosis, such as angiotensin-converting enzyme (ACE) inhibitors, angiotensin receptor blockers (ARBs) and some lipid-lowering drugs (25).

In fact, microRNAs can be a good indicator of cardiac fibrosis. In the presence of fibrosis, there is a downregulation in miR-24 expression (5), while miR-21 expression is upregulated (26). Actually, The miR-21 plays a crucial role in the promotion of fibrosis, as its antagonist has been shown to mitigate the extent of fibrotic development (27). The expression of miR-21 in macrophages modulates intercellular communication between macrophages and fibroblasts, thereby facilitating fibroblast-to-myofibroblast transition and exacerbating cardiac fibrosis (28). In addition, delivery of cell or tissue-specific anti-miR-29 can effectively suppress Wnt signaling and enhance the occurrence and progression of cardiac fibrosis (29). miR-214-3p exerts an inhibitory effect on cardiac fibrosis through downregulation of *NLRC5* gene expression and inhibition of the TGF-β1/Smad3 signaling pathway (30). The miR-126-5p can serve as a highly sensitive biomarker for the identification of sudden cardiac death resulting from coronary artery disease (31). The overexpression of miR-126-3p suppresses TGF-β-induced endothelial-to-mesenchymal transition (EndMT) and attenuates fibroblastogenesis, thereby ameliorating fibrosis (32). In summary, microRNAs can regulate the transdifferentiation of cardiac fibroblasts through intercellular signaling and transcription, which is expected to be a highly specific therapeutic target for cardiac fibrosis.

## 3 Exosome-borne miR-126 is a key factor in ameliorating cardiac fibrosis

### 3.1 Exosomes ameliorate cardiac fibrosis

Exosomes are widely distributed throughout the human body, present in almost all bodily fluids, and secreted by the majority of cells. They originate from the invagination of late endosomal membranes, resulting in the formation of numerous intraluminal vesicles that are subsequently released into the extracellular space through plasma membrane fusion. During this process, a multitude of proteins and primitive cell cytoplasm become encapsulated within these invaginated membranes to form intraluminal vesicles. Therefore, exosomes are believed to encompass primitive cellular proteins, lipids, and nucleic acids (33). Several studies have identified the presence of mRNAs and microRNAs within exosomes, with certain exosomes exhibiting elevated levels of microRNAs expression compared to their parent cells. These RNAs molecules can be transferred between cells via exosomes, facilitating the regulation of protein synthesis in recipient cells through a process known as intercellular shuttling of exosomal RNAs (34, 35). Exosomes are typically internalized by recipient cells through endocytosis, fusion with the plasma membrane, or receptor-ligand interactions (36–39). Therefore, exosomes can function as mediators of intercellular communication and pathways for modulating the expression of diverse intracellular RNAs and proteins, thereby exerting precise biological effects.

Exosomes are generally classified according to their cell origin, and exosomes from different sources have different effects. Studies have shown that exosomes can exhibit molecular characteristics similar to those of the original cells (40). For example, cardiovascular endothelial cell-derived exosomes carrying miR-214, miR-10a, miR-144, and miR-126 promote endothelial cell migration and angiogenesis (41), inhibit inflammatory responses (42), protect the heart (43), and inhibit apoptosis of vascular endothelial cells (44). Rejuvenation of cardiomyocytes and endothelial cells facilitated by miR-294-enriched exosomes derived from embryonic stem cells (45). Mesenchymal stromal cell-derived exosomes transport miR-182 for the regulation of inflammation (46). Exosomes derived from adipose stem cells carrying miR-126 exhibit a reduction in infarct size (47). Exosomes derived from cardiomyocytes, carrying miR-222 and miR-143, promote angiogenesis (48). Exosomes derived from macrophages, carrying miR-155 and miR-148a, exert inhibitory effects on fibroblasts proliferation and modulate immune responses (49, 50). mesenchymal stem cell (MSC) derived exosomes carrying miR-19a, miR-23a-3p, miR-138-5p, miR-29, and miR-24 inhibit cardiomyocyte apoptosis (51), attenuate myocardial injury (52), inhibit apoptosis, slow down the progression of myocardial infarction (53), reduce inflammation, and inhibit fibrosis (54). Exosomes derived from cardiomyosphere-derived cells carrying miR-181b mitigate infarct size (55). Exosomes derived from endothelial progenitor cells carrying miR-126 mitigate infarct size and apoptosis (56) (Table 1). Therefore, exosomes from different sources play their own roles in cell proliferation, angiogenesis and immune regulation, but sometimes exosomes from different sources can also play similar roles. As mentioned above, exosomes from cardiovascular endothelial cells, mesenchymal muscle cells and mesenchymal stem cells are related to inflammation, which means that in the face of the same pathological damage, exosomes from different sources can also play similar roles.

**Table 1:** Protective effects of different cell exosomes on the heart.

Cells	Exosome Contents	Functions	References
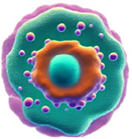 Endothelial cell	miR-214 miR-10a miR-144 miR-126	Promote angiogenesis Inhibit apoptosis and inflammation Protect the heart	(41)
			(42)
			(43)
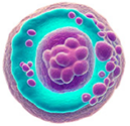 Embryonic stem cell	miR-294	Promotes survival of cardiomyocytes and endothelial cells	(45)
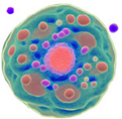 Mesenchymal stromal cells	miR-182	Regulate inflammation	(46)
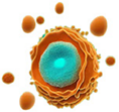 Fat stem cell	miR-126	Reduce infarct size	(47)
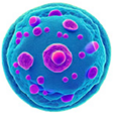 Cardiomyocyte	miR-222 miR-143	Stimulate angiogenesis	(48)
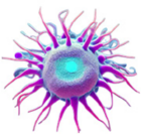 Macrophage	miR-155 miR-148a	Regulate immune response and fibroblast proliferation	(49)
			(50)

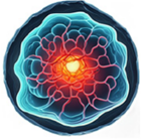 Mesenchymal stem cells	miR-19a miR-23a-3p miR-135-5p miR-29 miR-24 miR-126	Inhibit myocardial cell apoptosis Reduce myocardial injury Reduce inflammation Inhibit fibrosis	(51)
			(53)
			(52)
			(54)
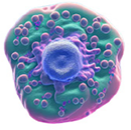 Cardiomyosphere-derived cells	miR-181b	Reduce infarct size	(55)
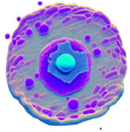 Endothelial progenitor cells	miR-126	Reduce cell apoptosis Reduce infarct size	(56)

Endothelial cell-derived exosomes, carrying miR-214, miR-10a, miR-144 and miR-126 promote angiogenesis, inhibit apoptosis and inflammation, and protect the heart. Embryonic stem cell-derived exosomes, harboring miR-294, promote viability in both cardiomyocytes and endothelial cells. Mesenchymal stromal cell-derived exosomes carry miR-182 to regulate inflammation. Fat stem cell-derived exosomes carrying miR-126 reduce infarct size. Cardiomyocyte-derived exosomes carrying miR-222, miR-143 stimulate angiogenesis. Macrophage-derived exosomes carrying miR-155 and miR-148a regulate immune responses and fibroblast proliferation. MSC-derived exosomes, carrying miR-19a, miR-23a-3p, miR-138-5p, miR-29, and miR-24, inhibit myocardial cell apoptosis, reduce myocardial injury, inflammation, and fibrosis. Cardiomyosphere-derived cell-derived exosomes carrying miR-181b reduce infarct size. Endothelial progenitor cell-derived exosomes, carrying miR-126 reduce infarct size and apoptosis.

Recent research has found that the protective effect of exosomes on the heart is largely achieved through the microRNAs carried by it. Exosomes-derived circ_0001785 can reduce endothelial cell damage through miR-513a-5p/TGFBR3 ceRNA network mechanism, thereby delaying atherosclerosis (57), exosomes secreted by mesenchymal stem cells pretreated with tanshinone IIA (TSA) carrying miR-223-5p reduced monocyte infiltration and enhanced angiogenesis to alleviate myocardial ischemia/reperfusion (58), mesenchymal stem cell-derived exosomes carrying miR-125a-5p can promote angiogenesis and attenuate fibroblasts proliferation and activation, thereby helping to improve cardiomyocyte apoptosis and inflammation (59). Therefore, exosomes can reduce inflammation and myocardial injury, inhibit the proliferation and activation of cardiac fibroblasts, and reduce the expression of collagen fibers by carrying microRNAs, thereby slowing down the occurrence and development of cardiac fibrosis.

### 3.2 miR-126 is a key factor in cardiac protection

The expression of miR-126 is predominantly observed in endothelial cells and endothelial progenitor cells (60). Conversely, miR-126-3p exhibits exclusive localization within the endothelial cells of the heart and kidney (32). Among the diverse repertoire of microRNAs, miR-126 plays a pivotal role in conferring cardioprotection. The miR-126 not only exerts pro-angiogenic effects, attenuates vascular inflammation, modulates autophagy, and mitigates endothelial cell apoptosis through targeted regulation of downstream proteins but also holds promise as a potential biomarker for prognostication and therapeutic monitoring in acute myocardial infarction (61, 62). Experimental evidence suggests that miR-126 derived from mesenchymal stem cells may enhance angiogenesis in infarcted myocardium through the AKT/ERK signaling pathway (63). Furthermore, miR-126 functions as a hypoxia-inducible target of HAT/HDAC, thereby facilitating cardioprotection through the selective activation of cell survival and proangiogenic pathways during ischemic conditions (64). Additionally, in vascular endothelial cells, miR-126 augments the VEGF (vascular endothelial growth factor) pathway, thereby facilitating angiogenesis through precise targeting and regulation of *Spred1* and *PI3KR2* genes (65). Additionally, the downregulation of miR-126 serves as a significant biomarker for the diagnosis of cardiovascular diseases. For instance, patients with coronary artery disease exhibit substantially reduced levels of miR-126 in their endothelial cells (66). Significantly reduced levels of plasma miR-126 and downregulation of serum miR-126-3p were observed in patients with myocardial infarction compared to healthy individuals (67, 68). Therefore, miR-126 plays a crucial role in maintaining normal cardiac function.

### 3.3 Exosomes are important carriers of miR-126 for cardiac protection

**Figure 1:**
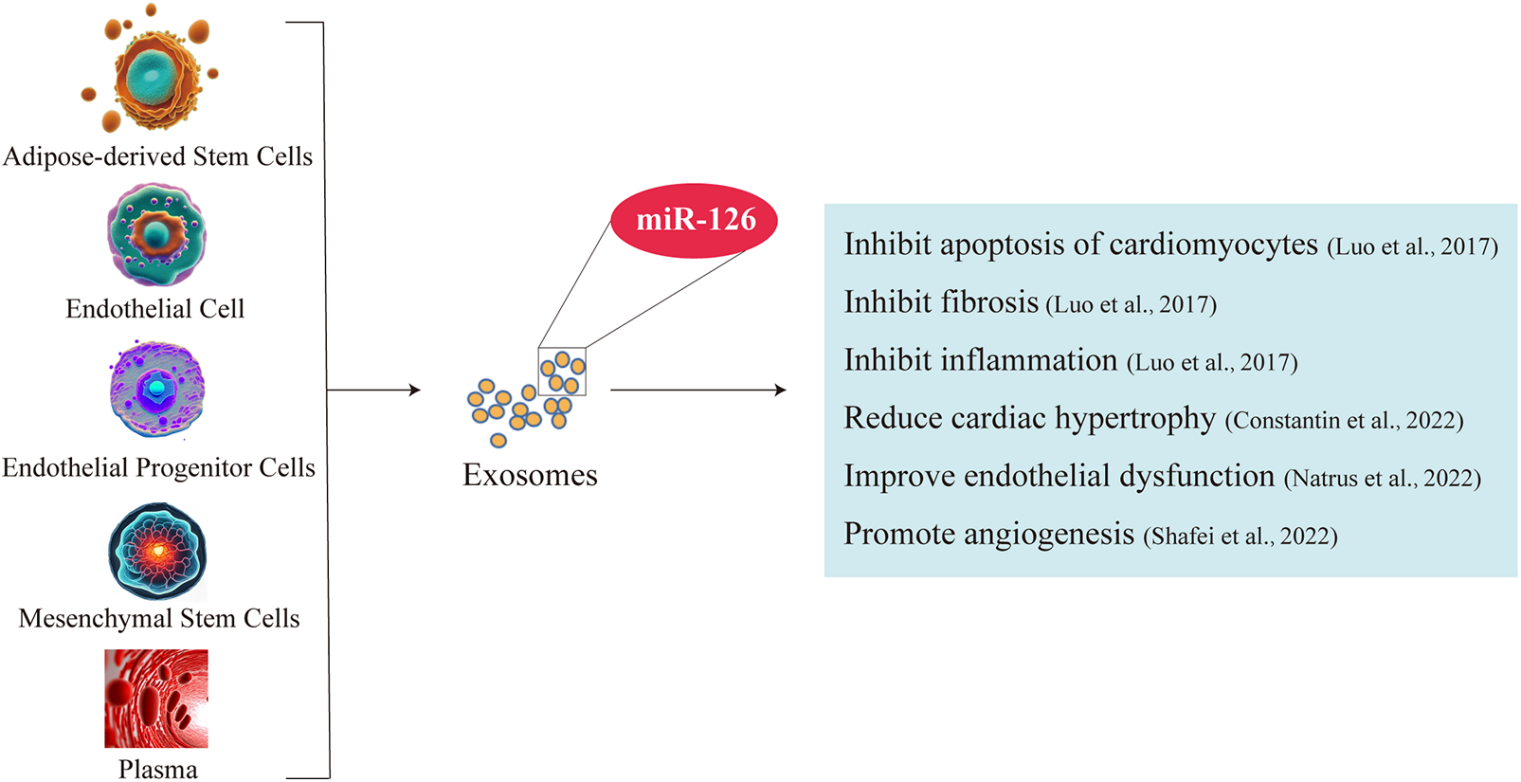
Exosomes are important carriers of miR-126 for its cardioprotective effects. Exosomes of fat stem cell, endothelial cell, endothelial progenitor cells, mesenchymal stem cells and plasma enriched in miR-126. Exosome-borne miR-126 inhibits cardiomyocyte apoptosis and cardiac inflammation, ameliorates fibrosis and endothelial dysfunction, and promotes angiogenesis.

After overexpressing miR-126 in exosomes secreted by adipose-derived Stem Cells (ADSCs), immunofluorescence analysis revealed the transfer of the upregulated miR-126 between ADSCs and H9C2 cells. This intercellular transfer not only promoted cell proliferation but also conferred protection against apoptosis, thereby mitigating damage to H9C2 cardiomyocytes and attenuating infarct area fibrosis. The ADSC-secreted exosomes containing miR-126 effectively suppressed cardiac inflammation by inhibiting the expression of pro-inflammatory factors IL1-β, IL-6, and TNF-a (47). Furthermore, stem cell-derived exosomes carrying miR-126 exhibit the potential to mitigate cardiac hypertrophy by impeding the inflammatory processes associated with it (69). Enhancement of cellular paracrine secretion through overexpression of plasma-derived exosome-borne miR-126 mitigates endothelial dysfunction and congestive heart failure (70). Moreover, exosome-mediated delivery of miR-126 plays a pivotal role in promoting angiogenesis and maintaining vascular integrity, thereby exerting a crucial influence on the prevention of heart failure and modulation of cardiac function (44). This suggests that exosome-mediated miR-126 not only inhibits cardiomyocyte apoptosis and cardiac inflammation, but also reduces fibrosis, alleviates endothelial dysfunction, and enhances angiogenesis. Therefore, exosomes are important carriers for miR-126 to play a cardiac protective role (Figure 1).

### 3.4 miR-126 regulates TGF-β/Smad3 signaling pathway to improve cardiac fibrosis

TGF-β serves as a pivotal regulator that orchestrates the transition from fibroblasts to myofibroblasts. This intricate process entails the binding of TGF-β to transmembrane receptor serine/threonine kinases (both type I and II), culminating in the formation of a complex. Subsequently, these complex triggers transphosphorylation of GS fragments within the type I receptor by the kinase activity of the type II receptor. Consequently, activation of the type I receptor ensues, leading to downstream phosphorylation events involving Smad2/3 (71). The phosphorylated molecules subsequently form a complex with Smad4 and translocate into the nucleus, facilitating their transcriptional activity. Within this context, Smad3 within the complex stimulates the transcription of pro-fibrotic molecules such as α-SMA and TIMP, thereby significantly contributing to fibroblast-to-myofibroblast transformation (72). The myofibroblasts secrete a large amount of extracellular matrix, which forms scar tissue after excessive deposition. This process reduces the compliance of the ventricular wall and eventually leads to cardiac dysfunction.

**Figure 2:**
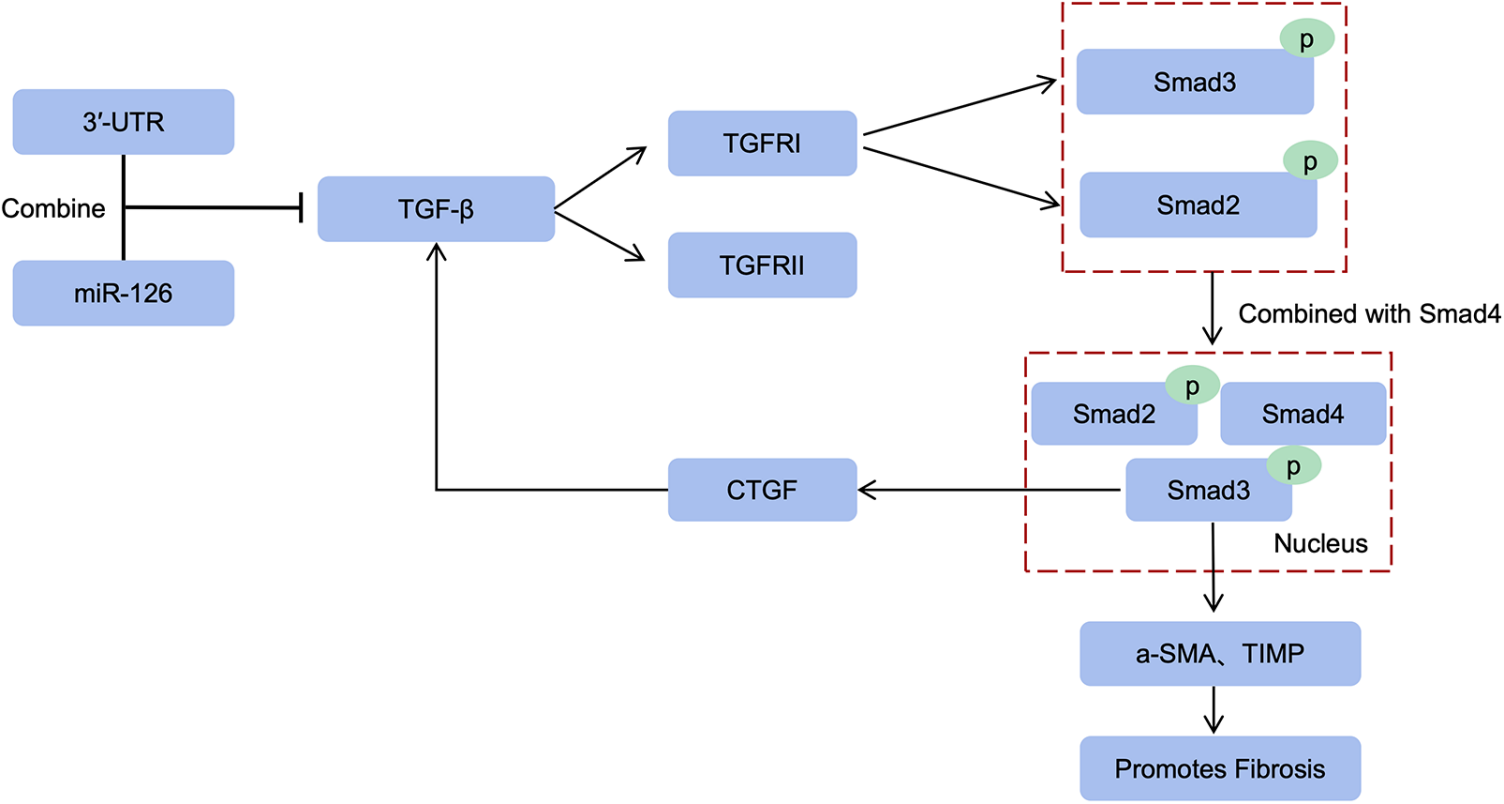
miR-126 inhibits the TGF-β/Smad3 pathway. miR-126 inhibits TGF-β translation thereby reducing type I receptor activation, and downstream Smad2/3 phosphorylation is also inhibited. Smad3 of the complex induces α-SMA, TIMP transcription to promote fibrosis. In addition, upregulation of CTGF expression promotes TGF-β-induced fibrosis, and Smad3 deficiency reduces fibrosis by decreasing CTGF expression.

It is noteworthy that miR-126 exerts translational inhibition on TGF-β by specifically binding to the 3′-untranslated region (3′-UTR) of TGF-β mRNA, thereby attenuating its expression and establishing TGF-β as a direct target of miR-126 (73). The levels of TGF-β are increased in cardiomyocytes with bidirectional knockout of miR-126 (74). Therefore, miR-126 not only reduced TGF-β expression, but also significantly downregulated the expression levels of TGF-β, Smad2 and Smad3, which resulted in a reduction in cardiomyocyte apoptosis and inhibition of cardiac fibroblasts activation (75). Up-regulation of connective tissue growth factor (CTGF) expression promotes TGF-β-induced fibrosis. In addition, Smad3 deficiency inhibits TGF-β-induced fibrosis by down-regulating the expression of connective tissue growth factor (76). Therefore, the TGF-β/Smad3 signaling pathway plays a crucial role in fibrosis, and miR-126 effectively inhibits it to improve cardiac fibrosis (Figure 2).

## 4 Exercise promotes exosome release to regulate cardiac function

Exercise exerts a profound positive impact on cardiovascular health. Experimental findings have demonstrated that during the post-infarction period, when ventricular contractility is reduced and cardiac function is compromised, eight weeks of continuous and intermittent exercise effectively enhance cardiac ejection fraction and overall function. Notably, the effect of intermittent exercise is particularly significant. This can be attributed to exercise's ability to enhance cardiac function by increasing blood delivery capacity to infarcted marginal zones (77). In comparison to aerobic exercise, “Hex” exercises (which encompass activities such as walking and gymnastics performed in warm water at temperatures ranging from 28 to 32 degrees Celsius) demonstrate a superior antihypertensive effect in individuals with persistent hypertension when conducted for 60 min, three times per week over a duration of 12 weeks (78). Moderate-intensity aerobic exercises, such as running, cycling, and dancing, have demonstrated efficacy in both the prevention and management of stage 1 hypertension. Furthermore, moderate dynamic resistance exercises like weightlifting can effectively reduce blood pressure to a significant extent (79). Long-term aerobic exercise effectively mitigates the risk of coronary heart disease by significantly reducing levels of apolipoprotein C3 and triglycerides (80).

Heart failure is a product of various cardiovascular diseases developed to the end stage, and exercise training can improve heart failure has been widely recognized (81). As an important pathological process of heart failure, can exercise have a good regulatory effect on cardiac fibrosis? We found that aerobic training plays an important role in attenuating cardiac fibrosis caused by vitamin D deficiency through its inhibitory effect on fibrotic factors and pathways (82), another study found that aerobic training combined with resistance exercise and vitamin D supplementation could reduce cardiac fibrosis by inhibiting TGF-β/Smads signaling pathway and regulating collagen fiber expression (83), running training, the most maneuverable aerobic exercise, still has a good improvement effect on cardiac fibrosis, for example, Ang II-induced cardiac fibrosis was attenuated in mice trained by running for 6 weeks (84), in a rat model of arthritis, individualized running training not only reduced fibrosis, but also alleviated the severity of arthritis (85). Moreover, exercise training can modulates the fibroblasts FGF21 factor and TGF-β1-Smad2/3-MMP2/9 signaling pathway to attenuate collagen fiber production (86). Exercise can also inhibits the induction of physiologic hypertrophy and myocardium by regulating cardiac microRNAs, and prevents cardiac fibrosis and dysfunction (87). To sum up, exercise can not only play a positive role in the prevention and treatment of cardiovascular diseases such as hypertension and coronary heart disease, but also better alleviate the pathological process of cardiac fibrosis. Aerobic exercise is often used as an example, which may be related to the significant effect of aerobic exercise on patients with heart failure. A meta-analysis has confirmed that aerobic training can reverse left ventricular remodeling in clinically stable heart failure patients (88). A comprehensive review suggests that a single bout of exercise can enhance exosome secretion, exhibiting variations in the metabolic kinetics of exosome production and clearance among individuals with different exercise intensities. Furthermore, prolonged and sustained exercise does not significantly affect the size and concentration of circulating exosomes, however, it selectively augments the release of exosomes from specific cellular sources. Moreover, long-term exercise exhibits a positive health-promoting effect by modulating both the type and quantity of produced exosomes, achieved through precise regulation of specific exosomal expression patterns (89). In conclusion, variations in exercise modes and intensities have been observed to influence the release of exosomes as well as their cargo of diverse microRNAs. Nonetheless, it is evident that exercise promotes exosome secretion, facilitating intercellular communication and regulation through microRNAs signaling. Moreover, exercise has the potential to modulate cardiovascular function via microRNAs encapsulated within exosomes (Table 2). For example, physical exercise enhances the levels of miR-126 in exosomes derived from circulating Endothelial Progenitor Cells (EPCs), thereby improving outcomes in mice with ischemic stroke (56). Exercise also promotes the release of plasma exosomes harboring miR-342, which effectively suppresses cardiomyocyte apoptosis and confers cardioprotection to the heart (97). Moreover, it effectively attenuates cardiac fibrosis by suppressing the MMP9 gene expression via exosomes derived from cardiomyocytes (98). Therefore, exosomes can carry microRNAs to play specific roles, and exercise can effectively stimulate the secretion and release of exosomes in cells, thus exerting their biological effects.

**Table 2:** Different exercise modalities promote the release of microRNAs from exosomes.

Exercise modalities	Source of exosomes	microRNAs	References
Endurance exercise	Plasma	miR-181a-5p	(90)
Marathon	Plasma and urine	hsa-mir-582-3p hsa-mir-199a-3p	(91)
Inertial resistance training	Hematology	miR-206 miR-146a	(92)
Physical exercise	Muscle and plasma	miR-21-5p miR-134-3p miR-107	(93)
Acute endurance exercise	Sweat cell	miR-21	(94)
Acute resistance training	Plasma	miR-532 miR-133a	(95)
Two consecutive rounds of muscle damage exercise	Hematology	miR-1 miR-206	(96)
Physical exercise	Endothelial progenitor cells	miR-126	(8)
Permanent exercise	Plasma and endothelial cells	miR-342-5p	(97)

Endurance exercise promotes the release of miR-181a-5p from plasma exosomes. Marathon promotes the release of hsa-mir-582-3p, hsa-mir-199a-3p from plasma and urinary exosomes. Inertial resistance training promotes the release of miR-206, miR-46a from blood exosomes. Physical exercise promotes the release of miR-21-5p, miR-134-3p, and miR-107 from muscle and plasma exosomes. Acute endurance exercise promotes the release of miR-21 from exosomes in sweat cells. Acute resistance training promotes plasma exosomes release of miR-532, miR-133a. Two consecutive rounds of muscle-damaging exercise promoted the release of miR-1 and miR-206 from exosomes in the blood. Physical exercise promotes miR-126 release from exosomes in endothelial progenitor cells. Permanent exercise promotes exosomes release from plasma and endothelial cells miR-342-5p.

## 5 Exercise promotes endothelial progenitor cell-derived exosomes release and miR-126 upregulation to improve cardiac fibrosis

**Figure 3:**
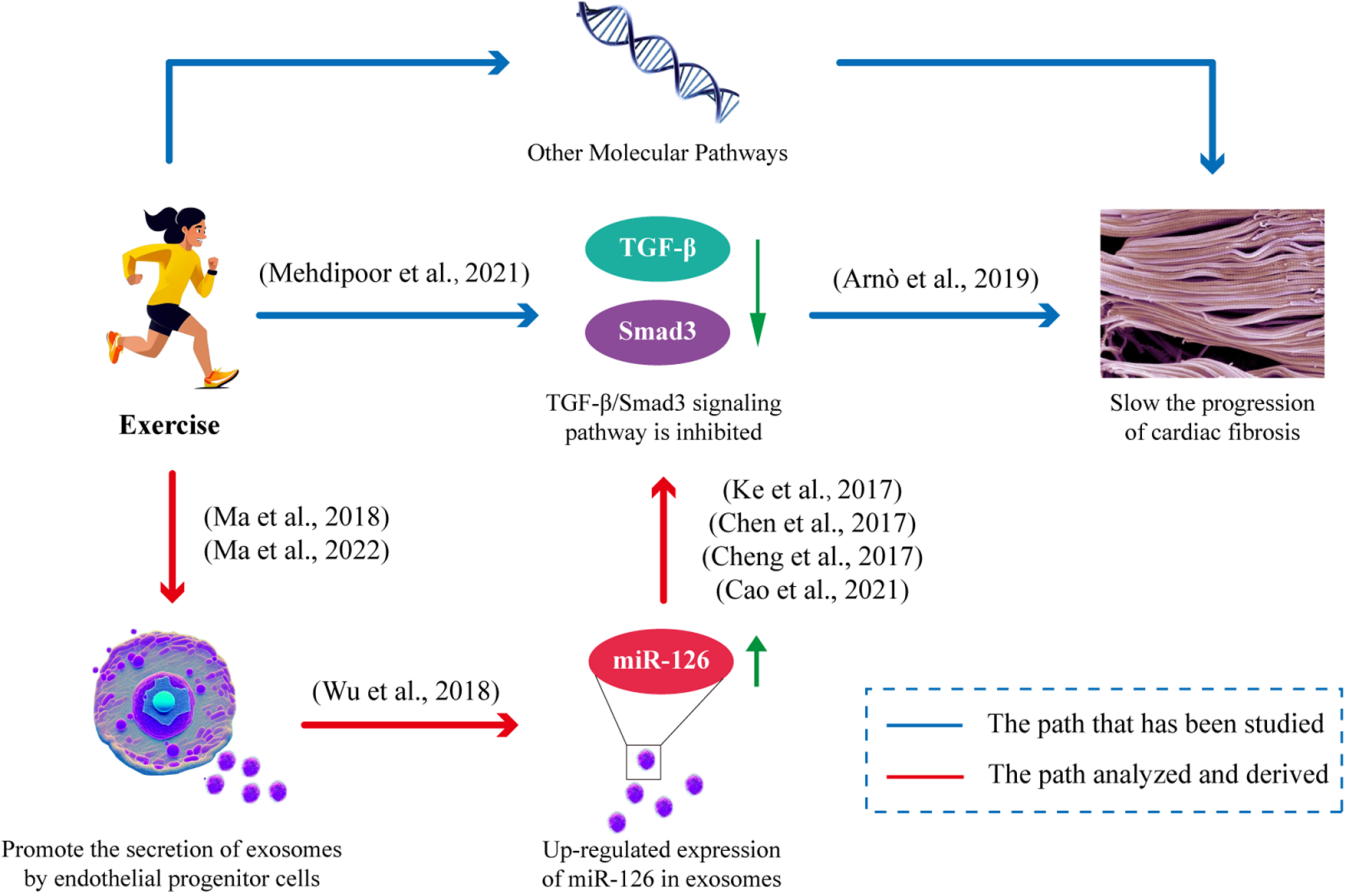
Exercise improves cardiac fibrosis mitigation by facilitating the release of EPC-Exos and modulating miR-126 expression. Exercise stimulates the secretion of EPC-Exos, thereby augmenting the levels of its constituent, miR-126. miR-126 effectively suppresses the TGF-β/Smad3 signaling pathway, consequently impeding cardiac fibrosis progression.

Endothelial Progenitor Cells (EPCs) inherently possess robust repair and regeneration capabilities. In comparison to exosomes derived from alternative sources, EPC-derived exosomes offer distinct advantages due to their notable enrichment in miR-126, a pivotal factor in mitigating cardiac injury. Furthermore, it has been demonstrated that moderate exercise can elevate the release of EPC-derived exosomes. Studies have demonstrated that endurance aerobic exercise can enhance the level of miR-126 in circulation and myocardium, augment EPCs, EPC-derived extracellular vesicles (EPC-Exos), and their cargo of miR-126 to exert a cardioprotective effect, with moderate intensity exercise being more efficacious than low intensity exercise (8, 62). Furthermore, the levels of miR-126 in EPC-derived exosomes (EPC-Exos) are positively influenced by exercise intensity, and these exosomes exhibit a comparable molecular signature to that of endothelial progenitor cells (EPCs) (8, 40). Exercise not only enhances the expression of miR-126 within endothelial progenitor cell-derived exosomes (EPC-Exos), but also augments the migratory capacity of EPCs when miR-126 is overexpressed in exosomes (40), thereby facilitating their transportation to the heart and exerting protective effects. These exosomes secreted by EPCs exhibit cardioprotective effects by stimulating cardiac fibroblasts, upregulating the expression of endothelial cell-specific markers, and downregulating proteins associated with fibrosis (99). The exosomes secreted by endothelial progenitor cells (EPCs) additionally enhance peri-infarct myocardial angiogenesis and improve hemodynamic function following infarction (100). Additionally, they provide cardiovascular protection through their anti-inflammatory and antioxidant properties, as well as their ability to modulate apoptosis and gene expression (101–103), all while exhibiting high levels of miR-126-3p and miR-126-5p (104). Importantly, studies have confirmed that exosomes effectively reach the heart and are efficiently internalized by neighboring and distant cells, thereby facilitating the regulation of receptor cells (105, 106). Experimental evidence has demonstrated the substantial uptake capacity of exosomes by heart cells, which may occur through direct interaction between transmembrane proteins on the exosome and signaling receptors in cardiomyocytes, or via fusion of the exosome with the plasma membrane of the cardiomyocyte to facilitate cargo transport, such as miR-126, into the cardiomyocyte (107). The TGF-β/Smad3 signaling pathway has been identified as a pivotal mediator of the cardiac fibrotic response (108). miR-126 attenuates cardiac fibrosis by suppressing the protein expression of TGF-β, Smad2, and Smad3 (73–75) (Figure 3). In conclusion, exercise can stimulate the secretion and release of endothelial progenitor cell exosomes (EPC-Exos), and inhibit TGF-β/Smad3 signaling pathway by upregulating the expression of miR-126 in exosomes to slow down the level of cardiac fibrosis.

## 6 Conclusions

miR-126 plays a key role in cardiac protection, and EPC-Exos also carry a large amount of miR-126. It was found that exercise can effectively stimulate the secretion and release of exosomes by EPCs, and up-regulate the expression of miR-126 in exosomes, which not only reduces the apoptosis of cardiomyocytes, but also improves cardiac fibrosis by targeting TGF-β. Therefore, miR-126 carried by EPC-Exos is expected to be a potential regulatory factor for the prevention and treatment of cardiac fibrosis. Exercise can play a protective role in the heart by regulating the release of exosomes and the expression of contents, which clarifies a new mechanism of exercise promoting cardiac rehabilitation. However, further investigation is warranted to elucidate the precise relationship between exercise intensity, duration, and exosome release quantity, as well as microRNAs expression. Continued research and study are necessary for the clinical application of anti-cardiac fibrosis methods such as exercise or exogenous exosome administration. The potential of exercise-mediated exosome therapy for cardiac fibrosis may emerge as a focus in future cardiovascular disease research.
